# A Humanized Diet Profile May Facilitate Colonization and Immune Stimulation in Human Microbiota-Colonized Mice

**DOI:** 10.3389/fmicb.2020.01336

**Published:** 2020-06-19

**Authors:** Isabel Moreno-Indias, Randi Lundberg, Lukasz Krych, Stine Broeng Metzdorff, Witold Kot, Dorte Bratbo Sørensen, Dennis Sandris Nielsen, Camilla Hartmann Friis Hansen, Axel K. Hansen

**Affiliations:** ^1^Section of Experimental Animal Models, Department of Veterinary Animal Sciences, Faculty of Health and Medical Sciences, University of Copenhagen, Copenhagen, Denmark; ^2^Unidad de Gestión Clínica de Endocrinología y Nutrición del Hospital Virgen de la Victoria, Instituto de Investigación Biomédica de Málaga (IBIMA), Universidad de Málaga, Málaga, Spain; ^3^Centro de Investigación Biomédica en Red de Fisiopatología de la Obesidad y la Nutrición, CIBERobn, Madrid, Spain; ^4^Internal Research and Development, Taconic Biosciences, Lille Skensved, Denmark; ^5^Department of Food Science, Faculty of Science, University of Copenhagen, Copenhagen, Denmark; ^6^Department of Plant and Environmental Science, University of Copenhagen, Copenhagen, Denmark

**Keywords:** gastrointestinal microbiome, mice, diet, fecal microbiota transplantation, lymphocytes, flow cytometry, gene expression

## Abstract

**Background:**

In spite of the importance of the use of gnotobiotic mice for human fecal transfer, colonization efficiency and immune stimulation after human microbiota inoculation in mice are poorly studied compared to mouse microbiota inoculation. We tested the colonization efficiency and immune responses in mice bred for one additional generation after inoculating the parent generation with either a human (HM) or a mouse microbiota (MM). Furthermore, we tested if colonization efficiency and immune stimulation could be improved in HM-colonized mice by dietary approaches: if these were fed a diet closer to the human diet either in its sources of animal fat and protein [the “animal source” (AS) diet] or in its proportions of macronutrients from the normal sources of a mouse diet [the “human profile” (HP) diet].

**Results:**

Although significantly lower in mice with a human microbiota (30–40% vs. 61–70%) the colonization efficiency was significantly higher in HM mice fed the HP diet (40%), and in MM mice fed AS (70%). The microbiota of mice fed HP was comparable to the microbiota of mice fed a standard rodent chow, while the microbiota of mice fed the animal source diet (AS) clustered separately. Mice inoculated with mouse fecal matter had significantly more CD4^+^ T cells and *Cd4* expression and significantly fewer regulatory T cells (Tregs) and *FoxP3* expression than human microbiota inoculated mice, but cell proportions differences were mostly apparent between mice fed the AS diet. Mice fed the HP diet had significantly higher expression of *Cd8a*.

**Conclusion:**

It is concluded that a diet with a humanized profile could support the establishment of a human microbiota in mice, which will, however, still elicit a lower colonization efficiency compared to mice inoculated with a mouse microbiota.

## Introduction

Animals colonized with human microbiota have been studied since the early days of gnotobiotics, the specialized technical field allowing the generation and housing of germ-free (GF) and defined microbiota animals. Initial work mostly focused on gnotobiotic animals monoassociated with human pathogenic bacterial strains (Gibbons et al., 1964; Sasaki et al., 1970; Moberg and Sugiyama, 1979; Czuprynski et al., 1983). The last decades, coinciding with the development of high-throughput sequencing techniques and their use in mapping the human microbiome (Qin et al., 2010; Consortium, 2012), has seen a renewed boom in the utilization of gnotobiotic animals as tools for understanding the complex commensal microbiome and its role in health and disease. Human microbiota (HM) colonized mice are considered relevant translational model systems, but limitations of the concept have been emphasized (Hirayama and Itoh, 2005; Nguyen et al., 2015; Arrieta et al., 2016b). Besides obvious and known differences between the human and murine anatomy and physiology (Nguyen et al., 2015), the limitations primarily concern two aspects: the inability to fully capture the compositional donor community in the recipients (Chung et al., 2012; Wos-Oxley et al., 2012; Lundberg et al., 2020), and altered shaping of the recipient immune system (Chung et al., 2012; Arrieta et al., 2016a; Lundberg et al., 2020).

Nutrients are critically important in shaping the structure of host-associated microbial communities (Hooper et al., 2012). Alterations in gut microbiota due to shifts in the diet composition has been extensively studied (Hildebrandt et al., 2009; Kashyap et al., 2013; Zhang and Yang, 2016). However, HM colonized mice are often fed standard rodent diets after colonization. Mice harboring HM express a set of metabolites distinct from that of their mouse microbiota (MM) harboring counterparts (Marcobal et al., 2013; Zhang et al., 2014), and the gut microbiota can change rapidly with changes in the diet (David et al., 2013). Differences in human and laboratory animal diets have been mentioned as a likely part of the problem with HM colonized mice (Hirayama and Itoh, 2005) and rats (Licht et al., 2007). Indeed, Hirayama et al. (1994) found that *Bifidobacterium* spp. increased significantly after shifting the diet of HM colonized mice from standard chow diet to chow diet enriched with fibers in the form of wheat bran (Hirayama et al., 1994). Turnbaugh et al. (2009) demonstrated how HM colonized mice switched from chow diet to a purified high-fat/high-sugar diet, the so-called Western diet, displayed rapid and significant changes in the microbiome in response to the diet change. Standard rodent chow diets differ substantially from what is considered a common human diet as they are often solely based on plant sources such as soy and cereal, sometimes with a fish meal part added. Additionally, chow diets often have a higher energy contribution from carbohydrates and lower contribution from fats compared to what is recommended for a human diet (Efsa, 2010; EFSA Panel on Dietetic Products Nutrition and Allergies, 2010). Indeed, dietary fat has been suggested to be the major contributing factor for microbiota modification (Agans et al., 2018).

Moreover, altered immunological shaping of HM colonized mice is sporadically reported in the literature but has not been extensively described. Compared to conventional mice, the small intestine of HM colonized mice lack expression of major histocompatibility complex class II molecules, had few IgA-producing cells, and an altered composition of IELs – all similarly observed in GF mice (Imaoka et al., 2004). More recently, HM colonized Swiss Webster mice were shown to resemble GF mice by having low numbers of T cells in the small intestinal lamina propria, PP and MLN, in addition to low expression of the antimicrobial peptide REGIIIγ in ileal tissue, and few DCs in PP and MLN (Chung et al., 2012). Moreover, we recently confirmed low expression of genes encoding for cluster of differentiation (CD)8a, CD4, FOXP3, and REGIIIγ in the ileum of HM colonized C57BL/6NTac mice on chow diet, possibly due to decreased stimulation of TLR by the HM (Lundberg et al., 2020).

Thus, there is little knowledge on whether a more humanized microbiota composition can be achieved by feeding the recipients a diet which in its composition is closer to the human diet from the moment of colonization. Therefore, we hypothesized that colonization efficiency and immune stimulation could be improved in HM colonized mice with a dietary approximation. In order to test our hypothesis, we have tried two different approaches: a diet closer to the human diet either in its sources of animal fat and protein or in its proportions of macronutrients from the normal sources of a mouse diet.

## Materials and Methods

### Mice, Inoculation, and Housing

**FIGURE 1:**
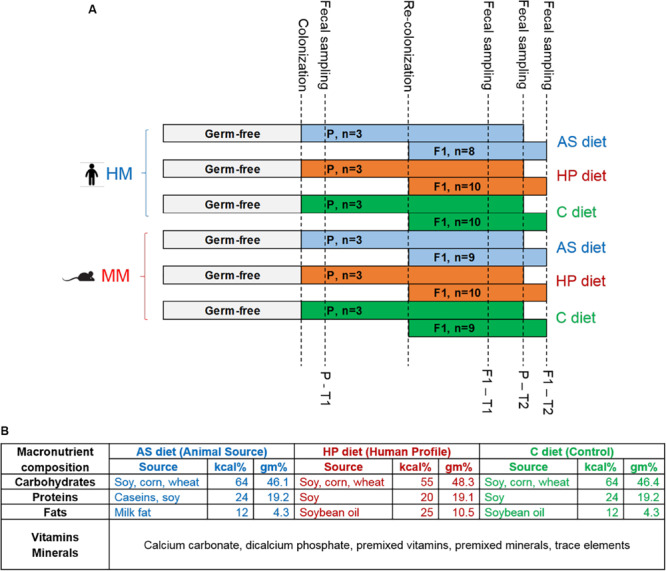
**(A)** Study design. Timeline showing the six experimental groups. Germ-free parent (P) mice were colonized with human microbiota (HM) or mouse microbiota (MM) inoculum when 6 weeks old and received animal source (AS) diet, human profile (HP) diet or control (C) diet from the day of arrival in our facility. After 1 week of acclimatization, P mice were bred. When F1 pups were born, the microbiota colonization was re-inforced by applying the inoculum to the abdomen of the lactating dams. Fecal samples for 16S rRNA gene sequencing analysis were sampled at two time points (T1, T2) for P and F1, respectively: P-T1, 1 week post-colonization, or 7 weeks of age; P-T2, 10 weeks post-colonization, or 16 weeks of age; F1-T1, 4 weeks of age; F1-T2, 6 weeks of age. **(B)** Macronutrient composition of the three diets used in the study. The C diet was Altromin #1320 maintenance diet for rats and mice, and the AS and HP diets were based on the C diet recipe. Kcal% = percentage of kilocalories coming from carbohydrates, proteins, and fats. Gm% = percentage in grams of carbohydrates, proteins, and fats.

Twelve female and six male GF C57BL/6NTac mice (Taconic Biosciences, Germantown, MD, United States), hereafter referred to as Parental mice (P), arrived at the SPF facility of University of Copenhagen, Frederiksberg, Denmark when 6 weeks old. On the day of arrival (Day 0), P mice were divided into two groups and colonized by oral gavage (50 μl/mouse) with a human or murine gut microbiota suspension (inoculates, Figure 1A) and socially housed according to sex. The inoculums were prepared by homogenizing human (500 mg of fecal material) or pooled mouse feces (from 9 mice, male and female) in sterile 25% glycerol (Merck Millipore, Darmstadt, Germany), which were frozen at −80°C until use. The donor mice were six male and three female C57BL/6NTac mice aged 10 weeks housed in the same SPF barrier as the recipient mice but originally obtained at 6 weeks of age from a Murine Pathogen Free (MPF^TM^) barrier (Taconic Biosciences, Ll. Skensved, Denmark). The donor mice were fed the same chow diet used as the C in our study (see description below). The human donor was a non-vegetarian omnivorous 36 years old male with a body mass index within the normal range (18.5–24.9 kg/m^2^). The diet of the human donor corresponded to a standard diet based on EFSA specifications (Efsa, 2010), and with no history of antibiotic treatment for at least 12 months before submitting the fecal sample to the study. The human donor was serologically screened negative for Hepatitis A, B and C, *Treponema pallidum*, HIV-1, and HIV-2. The human fecal sample was screened negative for *Helicobacter pylori*, *Salmonella*, *Shigella*, *Yersinia*, *Campylobacter*, *Clostridium difficile*, *Aeromonas*, *Plesiomona*s, *Vibrio*, pathogenic *Escherichia coli* strains, *Cryptosporidium*, *Giardia*, helminths and rotavirus, and additionally for the presence of *Proteus sp.*, *Klebsiella oxytoca*, *Klebsiella pneumoniae*, *Citrobacter rodentium*, *Staphylococcus aureus*, and *Pseudomonas aeruginosa*. Donor and recipient mice had identical local cage environment: Cages were polycarbonate Eurostandard Type III (Tecniplast, Varese, Italy), bedding was Tapvei aspen chips, and the mice had disposable Smart Home shelters, Mini Fun Tunnels, Enviro-Dri^®^ and Nestlets nesting material and Tapvei aspen size S gnawing blocks (all items purchased from Brogaarden, Lynge, Denmark). Ventilation was 15–20 air changes/hour, temperature was 20–24°C, relative humidity was 55% ± 10, and the light was on 6 a.m.–6 p.m. Recipient mice were housed in open-top cages in two ventilated cabinets (Scantainer^TM^, Scanbur, Karlslunde, Denmark) dedicated to human microbiota (HM) and mouse microbiota (MM) colonized mice respectively, and within each microbiota group fed three different diets as described below. Sterile water (Aqua B. Braun, B. Braun Denmark, Frederiksberg, Denmark) *ad libitum* was provided in bottles. Before populating with mice, the cabinets were disinfected with Rodalon^®^ (Abena, Aabenraa, Denmark). Inoculation of mice, cage changes, and handling was done in the room by a person wearing clean personal protective equipment (gown, gloves, face mask, and hair cover) and the table was disinfected with Rodalon^®^. All equipment and materials used were clean, but not sterilized, as we previously observed that autoclaving of cages, water bottles, and other materials did not pose an advantage in colonization of antibiotic-treated mice compared to no sterilization of materials (Ellekilde et al., 2014).

After 1 week of acclimatization, 2:1 (females:male) breeding trios were made. When pups were born, the microbiota was reinforced by applying the inoculum suspension on the abdomen of the dams with a swab. In this manner, pups reinforced microbiota during suckling. The number of F1 pups per group concluding the study was 8–10 HM pups/diet group (hereafter referred to as HM mice), and 9–10 MM pups/diet group (hereafter referred to as MM mice). Males and females’ proportions were determined by natural birth from their corresponding trios of P in each group. Individual fecal samples were obtained by placing mice in empty, clean cages inside the Scantainer^TM^. After approximately 60 min, fecal pellets were collected with sterile forceps and stored in sterile 1.5 ml Safe-Lock Eppendorf^®^ microcentrifuge tubes (Buch & Holm A/S, Herlev, Denmark). Fecal samples were obtained from P mice at 7 weeks of age (= 1 week post-colonization, T1) and at termination when at 16 weeks of age (T2), and from F1 pups 4 weeks of age (T1) and at termination at 6 weeks of age (T2). Fecal samples were stored at −80°C until DNA isolation.

### Diets

The Altromin #1320 maintenance diet (Altromin, Lage, Germany) for rats and mice was used as control (C) diet. In the human profile (HP) diet, Altromin changed the energy contribution from the macronutrients to match the recommended energy contribution proportions for humans ^31,32^, but left the ingredient sources unchanged, i.e., it was based on vegetable (cereals and soy) sources only. In the AS, Altromin partly substituted the vegetable proteins with caseins and all vegetable fats with milk fat, whereas the energy contribution levels were corresponding to that of the C diet (Figure 1B and Supplementary Table S1). The compound inositol (considered within the Vitamin E family) and the fatty acids capric acid, butyric acid, heptanoic acid, and octanoic acid were exclusively present in the AS diet, while the fatty acids margaric acid, arachidic acid, eicosenoic acid, behenic acid, and lignoceric acid were exclusively present in the HP and C diet. Diets were irradiated before use by 25 kGy.

### Food Consumption

From weaning to sacrifice at 6 weeks of age, individual body weights (BW) and cage food consumptions were recorded weekly for the F1 generation. Diet efficiencies were calculated by the following indices: Average daily food consumption (ADFC; g of food/day) and feed efficiency (g of BW gain/g of food consumption).

### Glycated Hemoglobin (HbA1c) Measurements

The day before termination of the study, 1 μL blood sample was obtained by puncturing the lateral tail vein of the F1 generation (6 week of age), and HbA1c was measured on a DCA Vantage Analyzer instrument (Siemens Healthcare A/S, Ballerup, Denmark).

### Fluorescence-Activated Cell Sorting (FACS)

Immediately after mice were sacrificed, MLN and PP were placed in cold PBS. While on ice and moist at all times, single cell suspensions were made by squeezing the organs between the rough surfaces of two microscope slides. The cell suspensions were passed through 100 μm nylon filters (Filcon, BD Biosciences, Kgs. Lyngby, Denmark), spun down, re-suspended in PBS and transferred to 96-well plates. Cells were surface stained for CD4, CD3, CD11b, CD11c, CD103, and CD19, for 30 min using the appropriate antibodies (eBioscience, San Diego, CA, United States). Intracellular staining for FOXP3 was done after fixating the cells with Fixation/Permeabilization buffer (stock concentrate diluted 1:4 with diluent; eBioscience) for 30 min, whereafter Permeabilization Buffer (eBioscience) diluted 1:10 with MilliQ water was added for 30 min. Analysis was performed using an Accuri C6 flow cytometer (Accuri Cytometers, Ann Arbor, MI, United States).

### Tissue Gene Expression by qPCR

Sections of ileum and colon of approximately 0.5 cm were excised cranially to the ileocecal valve and caudally to the colocecal junction, respectively. PP were excised from the samples for use in FACS, which were then cleaned from luminal contents, stored in 0.5 ml of RNAlater (Sigma-Aldrich, Brøndby, Denmark) and after soaking for 24 h at +4°C transferred to storage at −80°C. Tissue was homogenized in 0.5 ml MagMAX^TM^ Lysis/Binding Solution Concentrate (AM8500, ThermoFisher Scientific, Waltham, MA, United States), 3.5 μl β-mercaptoethanol and approximately 0.6 g of glass beads <106 μm (Sigma-Aldrich, Brøndby, Denmark) using a FastPrep 24 instrument (MP Biomedicals, Santa Ana, CA, United States). Homogenates were stored at −20°C. Total RNA was extracted using the MagMAX-96 Total RNA Isolation kit (ThermoFisher Scientific, Waltham, MA, United States) according to the manufacturer’s instructions on a MagMAX^TM^ Express Magnetic Particle Processor (ThermoFisher Scientific, Waltham, MA, United States). cDNA was synthesized from ∼500 ng total RNA by using the High-Capacity cDNA Reverse Transcriptase kit (ThermoFisher Scientific, Waltham, MA, United States) according to the manufacturer’s instructions. Levels of messenger RNA (mRNA) were measured by qPCR of *Cd4* (Mm00442754_m1), *Cd8a* (Mm01182107_g1), *Foxp3* (Mm00475162_m1), *Itgax* (Mm00498698_m1), *Il1b* (Mm01336189_m1), *Il10* (Mm00439616_m1), *Il18* (Mm00434225_m1), *Il6* (Mm00446190_m1), *Tgfb1* (Mm01178820_m1), and *Tnf* (Mm00443258_m1) using TaqMan^®^ gene expression assays (ThermoFisher Scientific, Waltham, MA, United States) and TaqMan^®^ Fast universal PCR Mastermix (ThermoFisher Scientific, Waltham, MA, United States) on cDNA duplicates on a StepOnePlus instrument (ThermoFisher Scientific, Waltham, MA, United States) as previously described (Hansen et al., 2013). Where both duplicate C_T_ values were returned as undetermined, we artificially set the C_T_ value to 40. If one duplicate was undetermined, and the other was returned with a high C_T_ value (e.g., 35–36), we used the single C_T_ value. Actin beta [*Actb* (Mm00607939_s1)] was used as the endogenous reference gene for normalization of data by defining ΔC_T_ as C_T(target)_ – C_T_(reference). Relative quantification (RQ) was then calculated as 2−ΔΔC_T_, where ΔΔC_T_ = ΔC_T_(sample) −ΔC_T_(calibrator), and where the calibrator was the mean ΔC_T_ of samples from MM colonized C diet mice.

### DNA Isolation and Library Building for 16S rRNA Gene Amplicon Sequencing

DNA was extracted using the PowerLyzer^®^ PowerSoil^®^ DNA Isolation Kit (MO BIO Laboratories, Inc., Carlsbad, CA, United States). Fecal samples were disrupted by the FastPrep FP120 Cell Disrupter (QBiogen, MP Biomedicals, France, speed 5.5, 3 × 30 s). Hereafter the manufacturer’s instructions were followed. Concentration and quality of the fecal DNA were measured using a NanoDrop 1000 Spectrophotometer (ThermoFisher Scientific, Waltham, MA, United States). The V3 region of the 16S rRNA gene was amplified with primers including adapters for the Nextera Index Kit (Illumina, San Diego, CA, United States): NXt_388_F: 5′- TCG TCG GCA GCG TCA GAT GTG TAT AAG AGA CAG ACW CCT ACG GGW GGC AGC AG −3′ and NXt_518_R: 5′- GTC TCG TGG GCT CGG AGA TGT GTA TAA GAG ACA GAT TAC CGC GGC TGC TGG −3′. PCR reactions containing 12 μl AccuPrime SuperMix II (Life Technologies, Carlsbad, CA, United States), 0.5 μl of each primer (10 μM), 5 μl of genomic DNA (20 ng/μl), and nuclease-free water to a total volume of 20 μl were run on a SureCycler 8800 (Agilent, Santa Clara, CA, United States). Cycling conditions applied were: Denaturation at 95°C for 2 min; 33 cycles of 95°C for 15 s, 55°C for 15 s, and 68°C for 40 s; followed by final elongation at 68°C for 4 min. To incorporate adapters and indices, PCR reactions contained 12 μl Phusion High-Fidelity PCR Master Mix (ThermoFisher Scientific, Waltham, MA, United States), 2 μl corresponding to P5 and P7 primer (Nextera Index Kit), 2 μl PCR product and nuclease-free water for a total volume of 25 μl. Cycling conditions applied were: 98°C for 1 min; 13 cycles of 98°C for 10 s, 55°C for 20 s, and 72°C for 20 s; final elongation at 72°C for 5 min. The amplified fragments with adapters and indices were purified using AMPure XP beads (Beckman Coulter Genomic, CA, United States). Prior to library pooling amplicons were quantified using a Qubit fluorometer (Invitrogen, Carlsbad, CA, United States) and pooled in equal amounts followed by sequencing on NextSeq platform (Illumina, San Diego, CA, United States) using the V2, 2 × 150 bp pair-ended kit chemistry.

### 16S rRNA Gene Amplicons and Data Processing

The raw dataset containing pair-ended reads with corresponding quality scores were merged and trimmed using settings as previously mentioned (Zachariassen et al., 2017). Quantitative Insight Into Microbial Ecology (QIIME; v 1.8.0 and 1.9.1) open source software package (Caporaso et al., 2010) was used for subsequent analysis steps. Removal of chimeric reads from the dataset and constructing *de novo* Operational Taxonomic Units (OTU) were conducted using the UPARSE pipeline (Edgar, 2013). The Greengenes (13.8) 16S rRNA gene collection was used as a reference database (McDonald et al., 2012). Unweighted, generalized, and weighted UniFrac distance metrics were calculated from subsampled OTU tables (10000 reads/sample) and visualized with non-metric multidimensional scaling (NMDS) plots. The differences in ordination between categories were tested using Permutational Multivariate Analysis of Variance using Distance Matrices, “adonis” function from R package “vegan” (Oksanen et al., 2011). Alpha diversity measure expressed with an observed species (sequence similarity 97% OTUs) value was computed for rarefied OTU tables (10000 reads/sample) using the alpha rarefaction workflow.

### Statistical Methods

Colonization efficiency was calculated by counting the overlapping number of OTUs (rarefied OTU table) between recipient mice and the inoculates. The number of OTUs in the inoculates was normalized to 100%, and the fraction thereof was calculated for the recipient mice. All quantitative data were subjected to normality test by Anderson–Darling test or for small group sizes by Shapiro–Wilk test, and test for equal variances by Brown–Forsythe’s test [GraphPad Prism 8 (GraphPad Software, La Jolla, CA, United States)]. If not fulfilling criteria for parametric testing, data were ranked before further statistical tests. Difference in alpha diversity were determined using a *t*-test-based approach employing the non-parametric (Monte Carlo) method (999 permutations) implemented in the compare alpha diversity workflow in QIIME. The differences in taxa abundance between categories were estimated with a statistic framework: analysis of the composition of microbes (ANCOM) based on non-normalized, species level OTU-table (Mandal et al., 2015), and ANOVA based on subsampled (10000 reads/sample) species level OTU-tables. HbA1c data were analyzed in Minitab^®^18 Statistical Software (Minitab Ltd., Coventry, United Kingdom) by one-way ANOVA with Tukey’s multiple comparisons *post hoc* test. BW, ADFC and feed efficiency results were analyzed by a two-way repeated measures ANOVA with Sidak’s multiple comparisons *post hoc* tests in (GraphPad Prism) with “Diet” and “Time” as factors, after checking that “Sex” factor did not interfere (*p* > 0.05). All other quantitative data were tested by a general linear model multifactorial ANOVA with random factors “inoculation” and “diet” and fixed factor “sex” (for bacterial abundances and colonization efficiency “time”) to reveal impact of these three factors, and subsequently by a one-way ANOVA for the factor “diet” as well as for the factor “group” (Minitab). All analyses were performed on a 95% significance level. All *p*-values for differences in bacterial abundances were subjected to correction for false discovery rate (FDR) by a two-stage linear step-up procedure (Benjamini et al., 2006) (Graph Pad Prism 8) only regarding *P* < 0.008 as significant discoveries, which in relation to diet and time (three groups) were then subjected to one way ANOVA with Tukey’s *post hoc* tests (Minitab 18).

## Results

### The Human Inoculum Was More Diverse Than the Mouse Inoculum

**FIGURE 2:**
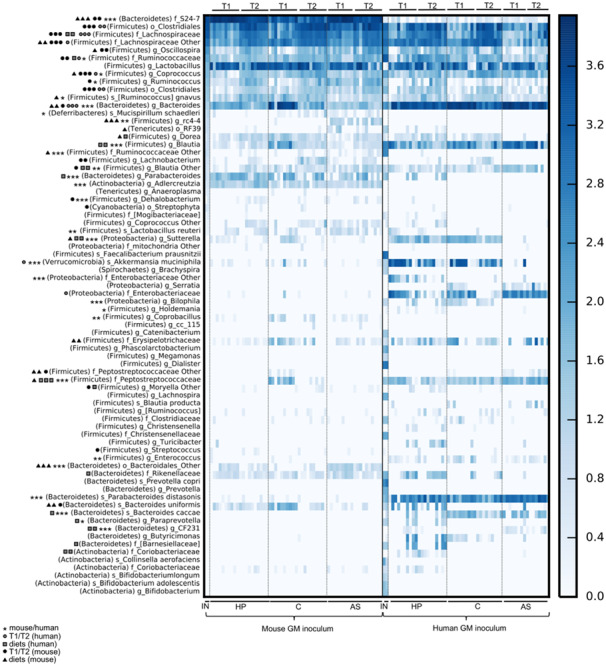
Overview of gut microbiota composition of mice born with a human or mouse microbiota and fed three different diets. Heat map presenting subsampled (10.000 reads/sample) OTU-table summarized to the species level. Symbols next to the taxonomic label indicate the level of significance of different abundances (1 symbol: *p* < 0.05, 2 symbols: *p* < 0.01, and 3 symbols: *p* < 0.001; FDR corrected p) and the categories subjected for testing with ANOVA. The lowest taxonomic level that a given OTU could be assigned to was listed (s: species, g: genus, f: family, o: order, c: class). The information about phylum is given in the brackets). T1, 4 weeks of age; T2, 6 weeks of age; IN, inoculum; AS, Animal source diet; HP, Human profile diet; C, Control diet; GM, gut microbiota.

The HM contained 62 identified OTUs. Firmicutes, i.e., members of Ruminococcaceae, Lachnospiraceae, and Christensenellaceae, of which some of the OTUs were quite well defined, such as *Dialister* spp., *Faecalibacterium prausnitzii*, *Blautia* spp., *Lachnospira* spp., and *Phascolarctobacterium* spp., dominated along with Bacteroidetes, i.e., Prevotellaceae including *Prevotella copri* and various other *Prevotella* spp. and Bacteroidaceae, including *Bacteroides* spp. The MM contained only 37 identified OTUs, and although it was dominated by the same phylae as the HM, the most abundant OTUs were S24-7, which were extremely low in the HM, and some less well defined Clostridiales spp. (Figure 2).

### Animal Source Diet and Human Profile Diet Supported Establishment of the Mouse Microbiota and Human Microbiota, Respectively

**FIGURE 3:**
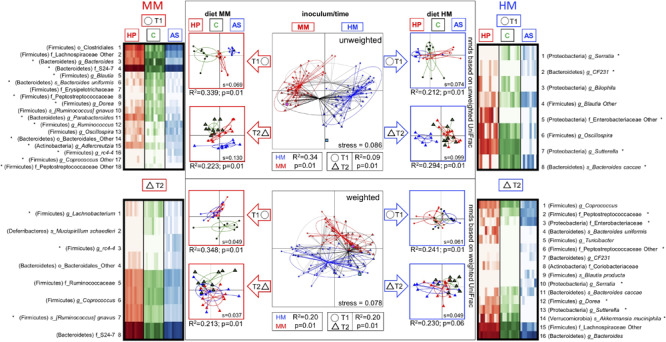
Gut microbiota composition of F1 mice and differences between mice on different diets. Differences in the gut microbiota composition of F1 mice due to: time (4 weeks of age = T1; 6 weeks of age = T2), diets (HP, C, AS), and inoculum (MM, HM) verified with 16S rRNA gene amplicon sequencing. NMDS plots based on unweighted (u) and weighted (w) uniFrac distance matrices show clear separation according to the inoculum source (MM = red; HM = blue), and time (T1 = circle; T2 triangle). The effect of diet (HP, C, and AS respectively: red, green, and blue) on microbial composition was further examined within each inoculum (red and blue arrows) and each time point (T1 = circle; T2 triangle) for which individual NMDS plots were generated. The heat maps depict taxa selected with ANCOM (at *p* = 0.1) to be different between the diets at each time point for both MM and HM. Significant differences (ANCOM *p* < 0.05) are marked with stars. Adonis results (*R*^2^ and *p* values) are presented below each NMDS plot. Donor inoculum samples of MM and HM are marked with magenta and blue squares, respectively. *S* values represent the stress of each NMDS ordination. HM, human microbiota; MM, Mouse microbiota; AS, animal source diet; HP, human profile diet; C, control diet.

We inoculated mice with HM and MM (P) and bred them to the next generation (F1). The colonization efficiency, i.e., the fraction of overlapping OTUs between recipient P and F1 mice and the inoculum they received, was significantly lower in HM mice compared to MM mice (*p* < 0.001; Table 1). Diet had a significant impact on colonization (*p* = 0.047; Table 1). In this manner, within HM the HP diet increased colonization compared to the AS diet (*p* = 0.009; Table 1) and borderline compared to the C diet (*p* = 0.053; Table 1), while within MM, the AS diet increased colonization compared to both the HP and the C diet (*p* = 0.000; Table 1). Time also had significant impact on the colonization efficiency (*p* = 0.002; Table 1), but this was mainly driven by a significantly lower colonization efficiency in the parent MM mice 1 week after colonization, which seemed to stabilize over time (*p* < 0.022; Table 1). Also, the parent HM mice had a significantly lower colonization efficiency 1 week after colonization compared to their offspring at 4 weeks of age (*p* = 0.044; Table 1). However, this was mostly due to a lower initial colonization efficiency for HM mice fed the C diet, and over time and generations they seemed to stabilize at a low colonization efficiency (Table 1). Some of the dominant OTUs in the HM inoculum, i.e., Firmicutes (*F. prausnitzii*, *Dialister*, Christensenellaceae, *Lachnospira*, and *Phascolarctobacterium*), one Spirochetes (*Brachyspira*) and two Bacteroidetes (*Prevotella* spp. and *P. copri*) did not colonize the recipients (Figure 3). On the other hand, 29 OTUs, which were not found in any of the inocula, for instance, *Bacteroides uniformis* in the MM mice, and Enterobacteriaceae and *Sutterella* in HM mice were subsequently identified in recipient mice (Figure 2).

**TABLE 1:** Colonization efficiency of mice colonized with human or mouse microbiota.

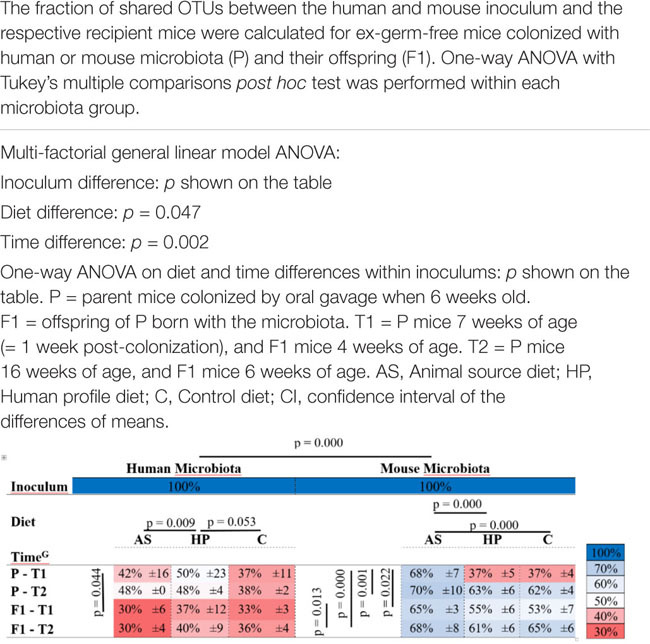

### The Animal Source Diet Resulted in the Most Distinct Microbiota Composition of Mice With Human and Mouse Microbiota

The microbiotas of HM and MM mice were clearly different, as illustrated by the NMDS plot based on unweighted (qualitative) and weighted (quantitative) UniFrac metrics, which displayed a clear separation between samples from HM- and MM colonized mice (Unweighted: *R*^2^ = 0.34, *p* = 0.01, Weighted: *R*^2^ = 0.20, *p* = 0.01; Figure 3). Samples from MM mice were closely associated to the MM inoculum, whereas samples from the HM colonized mice were more distant from the HM inoculum, though still closer to the HM inoculum than the MM samples. Especially the abundance of a range of Clostridiales and Bacteroidales species were significantly different between inocula.

Feeding the recipient mice different diets clearly resulted in differences in gut microbiota composition. *A. muciniphila* was lower in MM compared to HM mice, unless the HM mice were fed the AS diet, which reduced its abundance to a level comparable to the MM mice (Figure 2 and Table 2). The AS diet had a clear effect on the MM mice, as OTUs such as the unclassified “rc4-4” and RF39 genera of Firmicutes and Tenericutes, respectively, were more abundant in samples from the AS diet group (Figure 3 and Table 2).

**TABLE 2:** Significant differences in OTU abundances between recipient mice for a human (HM) and mouse inoculum (MM).

			**MM**			**HM**		
**Actinobacteria**	**p**	**q**	**HP**	**AS**	**C**	**HP**	**AS**	**C**
c__Coriobacteria;o__Coriobacteriales;f__Coriobacteriaceae;Other;Other	0.000	0.000	B	B	B	A	B	B
**Bacteroidetes**								
c__Bacteroidia;o__Bacteroidales;f__Bacteroidaceae;g__Bacteroides;s__	0.001	0.004	C	C	BC	AB	A	A
c__Bacteroidia;o__Bacteroidales;f__Bacteroidaceae;g__Bacteroides;s__caccae	0.000	0.000	B	A	B	B	B	B
c__Bacteroidia;o__Bacteroidales;f__Bacteroidaceae;g__Bacteroides;s__uniformis	0.000	0.000	A	BC	A	AB	BC	C
c__Bacteroidia;o__Bacteroidales;f__Porphyromonadaceae;g__Parabacteroides;s__	0.001	0.004	A	BC	AB	CD	DE	E
c__Bacteroidia;o__Bacteroidales;f__S24-7;g__;s__	0.000	0.000	A	A	B	C	C	C
c__Bacteroidia;o__Bacteroidales;Other;Other;Other	0.000	0.000	B	A	C	CD	D	D
**Firmicutes**								
c__Bacilli;o__Lactobacillales;f__Streptococcaceae;g__Streptococcus;s__	0.003	0.009	A	AB	AB	A	B	AB
c__Bacilli;o__Turicibacterales;f__Turicibacteraceae;g__Turicibacter;s__	0.000	0.000	B	B	B	A	B	B
c__Clostridia;o__Clostridiales;f__Lachnospiraceae;g__[Ruminococcus];s__gnavus	0.000	0.000	A	BC	A	BC	C	AB
c__Clostridia;o__Clostridiales;f__Lachnospiraceae;g__Anaerostipes;s__	0.003	0.009	B	A	B	B	B	B
c__Clostridia;o__Clostridiales;f__Lachnospiraceae;g__Blautia;Other	0.002	0.008	B	AB	AB	B	A	AB
c__Clostridia;o__Clostridiales;f__Lachnospiraceae;g__Blautia;s__	0.000	0.000	D	D	C	BC	A	AB
c__Clostridia;o__Clostridiales;f__Lachnospiraceae;g__Coprococcus;s__	0.000	0.000	BCD	D	C	CD	ABC	ABC
c__Clostridia;o__Clostridiales;f__Lachnospiraceae;g__Dorea;s__	0.003	0.009	AB	AB	A	AB	C	B
c__Clostridia;o__Clostridiales;f__Lachnospiraceae;Other;Other	0.000	0.000	BC	AB	A	A	C	A
c__Clostridia;o__Clostridiales;f__Peptococcaceae;g__;s__	0.003	0.009	B	A	B	B	B	B
c__Clostridia;o__Clostridiales;f__Peptococcaceae;g__rc4-4;s__	0.000	0.000	B	A	B	B	B	B
c__Clostridia;o__Clostridiales;f__Peptostreptococcaceae;g__;s__	0.000	0.000	C	C	B	AB	A	AB
c__Clostridia;o__Clostridiales;f__Peptostreptococcaceae;Other;Other	0.001	0.004	B	A	A	B	B	B
c__Clostridia;o__Clostridiales;f__Ruminococcaceae;Other;Other	0.003	0.009	BC	A	B	BC	C	BC
**Proteobacteria**								
c__Betaproteobacteria;o__Burkholderiales;f__Alcaligenaceae;g__Sutterella;s__	0.000	0.000	B	B	B	A	B	A
c__Gammaproteobacteria;o__Enterobacteriales;f__Enterobacteriaceae;g__Serratia;s__	0.000	0.000	C	C	C	C	A	B
c__Gammaproteobacteria;o__Enterobacteriales;f__Enterobacteriaceae;Other;Other	0.003	0.009	B	B	B	A	B	B
**Tenericutes**								
p__Tenericutes;c__Mollicutes;o__RF39;f__;g__;s__	0.001	0.004	BC	A	BC	B	C	C
**Verrucomicrobia**								
c_Verrucomicrobiae;o Verrucomicrobiales;f_Verrucomicrobiaceae;g_Akkermansia;s_muciniphila	0.001	0.004	B	B	B	A	B	A
**Unassigned**								
Unassigned;Other;Other;Other;Other;Other;Other	0.002	0.008	B	A	B	B	B	B

*Significant differences were revealed by multifactorial general linear model ANOVA on factors inoculum, diet and time (p), and *p*-values for all OTUs were subjected to false discovery rate (FDR) correction (q). If q was significant abudances were compared by a one-way ANOVA for the factor diet. Diets with different letters are significantly different. HP, Human profile diet; AS, Animal Source diet; C, Control diet.*

Time clearly affected microbiota profile. There was a significant separation between time points T1 and T2 (Unweighted: *R*^2^ = 0.09, *p* = 0.01, Weighted: *R*^2^ = 0.09, *p* = 0.01; Figure 3). Within each inoculum group, an early qualitative clustering of all three diets in the MM samples was revealed at T1 (Unweighted: *R*^2^ = 0.34, *p* = 0.01; Figure 3). The AS and HP diet samples also clustered together as determined by weighted Unifrac metrics, but separately from the C diet samples (Weighted: *R*^2^ = 0.34, *p* = 0.01; Figure 3). Clustering between diets (weighted Unifrac) was still significant at T2 in the MM samples (Unweighted: *R*^2^ = 0.22, *p* = 0.01), but the C and HP diets were closer to one another at T2 than at T1, whereas the AS diet was clearly distinct (Figure 3).

At T2, samples from all three diets were more closely associated to one another, though the clustering was still significant (Weighted Unifrac, *R*^2^ = 0.21, *p* = 0.01; Figure 3). Within the HM samples, there was a significant qualitative clustering at T1 (Unweighted Unifrac: *R*^2^ = 0.21, *p* = 0.01; Figure 3), although it was less distinct than observed for the MM samples. At T2, the HM samples displayed the same pattern as observed for the MM samples, i.e., that the HP and C diet approached one another and the AS diet formed a separate cluster (Unweighted: *R*^2^ = 0.29, *p* = 0.01). Weighted Unifrac metrics based on HM samples revealed a relatively close association, however, significantly different, between samples from all three diets at both T1 (Weighted: *R*^2^ = 0.24, *p* = 0.01) and T2 (Weighted: *R*^2^ = 0.23, *p* = 0.01, Figure 3).

The differential abundance analyses revealed that 13 OTUs differed between the diets in samples from MM colonized mice at T1, whereas only three OTUs differed between diets at T2, which could reflect the approximation of the HP and C diet with time. In samples from HM colonized mice, 5 OTUs differed between diets at T1, and 7 OTUs differed at T2, with the AS diet group displaying most differences, followed by the C diet group (Figure 3).

### Animal Source Diet Increased Regulatory T Cells in Mice With Human Microbiota

**FIGURE 4:**
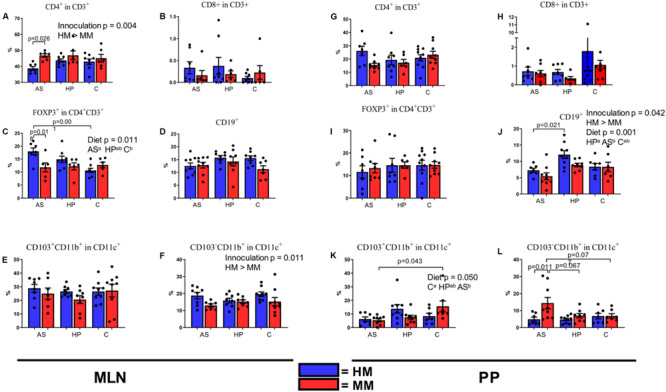
Populations of T cells **(A–C,G–I)**, dendritic cells **(E,F,K,L)**, and B cells **(D,J)** in mesenteric lymph nodes **(A–F)** and Peyer’s patches **(G–L)** of F1 mice on different diets. Cell populations were measured by fluorescence-activated cell sorting at 6 weeks of age. MLN, mesenteric lymph nodes; PP, Peyer’s patches; HM, human microbiota; MM, Mouse microbiota; AS, animal source diet; HP, human profile diet; C, control diet. Bars are SEM. Diets with different letters (a or b) are significantly different (*p*-values not shown).

To achieve research benefits from a mouse with a human microbiota, the immune stimulation of the microbiota should be comparable to normal conditions in a mammalian host. Intestinal DCs sample intestinal bacteria at the mucosal surface. These bacteria-laden DCs interact with B and T cells in the PP, inducing B cells to produce immunoglobulin A directed against intestinal bacteria (Coombes and Powrie, 2008). Finally, these bacteria are carried alive to the MLN. In order to observe if any of the experimental diets would improve the stimulation of the immune system by the HM inoculum, we analyzed the numbers of DCs, B cells, Th cells, and Tregs in MLN and PP of HM and MM mice. MM mice had a significantly higher percentage of CD4^+^ T cells in MLN (Figure 4A; *p* = 0.004), and this was especially driven by a significantly lower percentage in HM mice on the AS diet compared to MM mice (Figure 4A; *p* = 0.026). However CD4^+^ T cells in PP did not show differences (Figure 4G). The percentage of CD8^+^ T cells did not show any significant differences neither in inocula, nor in tissue (Figures 4B,H). Interestingly, the diet had a significant impact on the percentage of CD4^+^ Tregs (FOXP3^+^CD4^+^CD3^+^; *p* = 0.011; Figure 4C) with the AS diet inducing higher percentages (Figure 4C). In PP no such differences could be revealed (Figure 4I). There was no effect of inoculum or diet on B cells as assessed by CD19^+^ cells in MLN (Figure 4D), whereas in PP the percentage of B cells (CD19^+^) was higher in HM colonized mice on the HP diet compared to mice on the AS diet (*p* = 0.042; Figure 4J). Also, the percentage of B cells was significantly lower in AS fed mice compared to HP and C fed mice (*p* = 0.001; Figure 4J). There was no effect of sex on the FACS data.

### The Animal Source Diet Decreased the Number of Dendritic Cells in Mice With Mouse Microbiota

The HM inoculum stimulated the DC populations in MLN as assessed by staining for CD11b, CD11c, and CD103 better than the MM inoculum (*p* = 0.010; Figure 4E), but there was no significant diet impact on this in MLN (Figures 4E,F). In PP, there was significant effect of diet on the percentage of CD103^+^CD11b^+^ cells [tolerogenic or mainly a migratory DC population (Coombes et al., 2007)] (*p* = 0.050; Figure 4K), especially because the percentage of CD103^+^CD11b^+^ cells were borderline lower in AS fed mice (*p* = 0.055). Reversely, the percentage of CD103^–^CD11b^+^ cells (pro-inflammatory cytokine producing DC’s) was significantly higher in the PP of MM mice (*p* = 0.017; Figure 4L), which was especially pronounced in mice on the AS diet (p 0.011; Figure 4L).

### Mice With Human and Mouse Microbiota Differed in Gene Expression for Immune Cells, and the Human Profile Diet Increased the Expression of *Cd8a*

**FIGURE 5:**
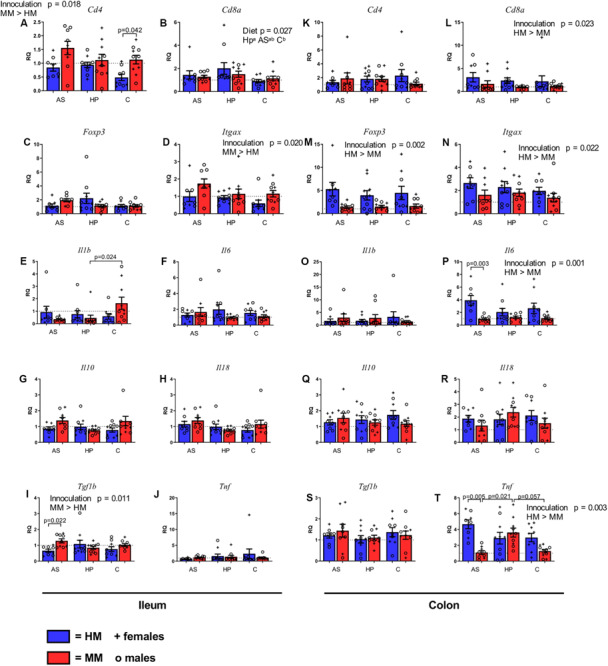
Gene expression of markers of T cells **(A–C,K–M)**, dendritic cells **(D,N)**, and cytokines **(E–J, O–T)** in ileum **(A–J)** and colon **(K–T)** of F1 mice on different diets. mRNA levels were measured by qPCR, reported as the relative quantification (RQ) compared to the mean of mice with mouse microbiota (MM) on control (C) diet. MM, Mouse microbiota; AS, animal source diet; HP, human profile diet; C, control diet. Bars are SEM. Diets with different letters (a or b) are significantly different (*p*-values not shown).

To reveal whether any of the experimental diets affected the number of T and DCs, we measured levels of mRNA of gene markers for these in ileum and colon tissues of HM and MM mice on the different diets. Ileal expression of *Cd4* was lower in HM mice compared to MM mice (*p* = 0.018; Figure 5A), although only significantly lower in mice fed the C diet (*p* = 0.042; Figure 5A). In colon there were no differences in *Cd4* expression (Figure 5K). About *Cd8a* expression, while there were no differences between inoculums in ileum, the HM mice had a higher expression of *Cd8a* compared to the MM mice in colon (*p* = 0.023; Figure 5L). However, the HP diet increased the ileal expression of *Cd8a* compared to the C diet (*p* = 0.029: Figure 5B). Neither diet nor the inoculum source seemed to influence the expression of *FoxP3* in ileum (Figure 5C), while in colon HM mice had higher *FoxP3* expression than MM mice (*p* = 0.002; Figure 5M). Expression of *Itgax* (activation of CD11c, i.e., DCs) was higher in MM mice compared to HM mice in ileum (*p* = 0.020; Figure 5D), while this was opposite in colon (*p* = 0.022; Figure 5N). Males had significantly higher ileal *Cd8a* expression (*p* = 0.021; data not shown).

### Mice With a Mouse Microbiota Have a Lower Expression of Some Pro-inflammatory Cytokine Genes

To reveal whether any of the experimental diets affected the level of inflammation, we measured levels of mRNA of gene markers for pro- and anti-inflammatory cytokines in ileum and colon tissue of HM and MM mice on the different diets. There were a few dietary effects on cytokine gene expression, and in general, the HM seemed to stimulate gene expression more in colon than in ileum, whereas the opposite was the case for the MM. In colon *Tnf* expression over all was higher in HM mice compared to MM mice (*p* = 0.003; Figure 5T), mostly because of the levels of MM mice on the AS diet. There were no differences in the expression of *Tnf* in ileum (Figure 5J). *Il1b* was expressed less in ileum of MM mice on the HP diet compared to MM mice on the C diet (*p* = 0.024, Figure 4E). No other differences were found in *Il1b* expression in ileon or colon (Figure 5O). In colon the expression of *Il6* was significantly higher in HM mice compared to MM mice (*p* = 0.001; Figure 5P), which was driven by a significantly higher expression in HM mice on the AS diet (*p* = 0.003, Figure 5P), but no differences were observed in ileum (Figure 5F). *Tgfb1* expression was higher in ileum of MM mice (*p* = 0.011; Figure 5I), which was mainly driven by a significant difference between AS fed mice (*p* = 0.022, Figure 5I). There were no differences in colon for *Tgfb1* (Figure 5S). *Il10* and *Il18* expressions did not differ between the groups in ileum or colon (Figures 5G,H,Q,R, respectively), and there were no sex related differences in cytokine gene expressions.

### Mice With Mouse Microbiota Consumed More of the Human Profile Diet

It is important to relate dietary results with diet consumption, growth and metabolism. Neither the inoculum nor the source or the diets affected the growth, as BW was the same in all groups from three to 6 weeks of age (Supplementary Figure S1A). The feed efficiency of the HP diet in the MM mice was lower compared to the other diets (*p* = 0.023 vs. AS; *p* = 0.018 vs. C; Supplementary Figure S1B), while HM mice seemed to exhibit the same feed efficiency for all three diets. Glycated hemoglobin (HbA1c), a measure of long-term blood glucose, was not different between diets groups, or between sexes (Supplementary Figure S1C).

## Discussion

We tested if colonization efficiency and immune stimulation could be improved in HM colonized mice if these were fed a diet closer to the human diet either in its sources of animal fat and protein (the AS diet) or in its proportions of macronutrients from the normal sources of a mouse diet (the HP diet). Colonization efficiency was better in MM mice compared to HM mice on all diets, but the HP diet improved colonization efficiency in HM mice. This indicates that HM colonization in mice is better helped by feeding the microbiota the right amount of nutrients rather than feeding them nutrients sourced like a human diet. The AS diet strongly improved colonization efficiency in MM mice, which is interesting but maybe not that surprising as wild mice in nature also are known to have a consumption of animal protein and fat (Whitaker, 1966; Khanam et al., 2016). However, although the HM was far more diverse than the mouse inoculum, this difference vanished in the recipient mice, due to a much lower colonization efficiency of the HM with a substantial loss of microbial taxa, especially belonging to phylum Firmicutes as previously shown (Raibaud et al., 1980; Wong et al., 1996; Imaoka et al., 2004; Chung et al., 2012; Wos-Oxley et al., 2012; Lundberg et al., 2020).

After 6 weeks of age, the gut microbiota of HP and C mice clustered together in both MM and HM mice. The AS diet induced HM formed its own distinct cluster. This was observed at 4 weeks of age already in the MM mice, whereas at this age it was less pronounced in the HM mice indicating a longer response time of the HM microbiota to the diet, perhaps because it needs more time to adjust to the host habitat, even though these mice were born with the microbiota. Diets might have been differences in nutrients which influence the growth and the stabilization of the gut microbiota. While absent in the HP or C diet, the AS diet was rich in milk fatty acids, such as capric, butyric, heptanoic, and octanoic acids. Dietary fat type may elicit a substantial and differential effect on the host-microbe relationship (Huang et al., 2013), e.g., a key ingredient affecting the crosstalk between gut microbiota and the host is dietary cholesterol (Kubeck et al., 2016), present in the AS diet in contrast to the two diets (HP and C) with vegetable fat. Diet did not affect glucose metabolism, and the mice grew similarly on the three diets, although the MM mice ate more of the HP diet, something that did not happen in HM mice. This indicates that the experimental diet did not induce major metabolic changes in the mice.

Interestingly, diets differently stimulated the immune system. As also observed by others (Chung et al., 2012; Lundberg et al., 2020), our HM mice did obtain a lower fraction of CD4^+^ T cells in the MLN and lower expression of the *Cd4* gene in ileum. However, it was mainly HM mice fed the AS diet, which had a very low fraction of CD4^+^ T cells, and as these also had significantly higher fractions of Tregs, their absolute numbers of Th cells were probably rather low. The difference in gene expression was also very pronounced between HM and MM mice fed the C diet, while the mice seemed more comparable, when fed the HP diet. The HM mice, especially if fed the HP diet, had higher fractions of B cells in PP, which is the main organ for B cell differentiation, and they had higher expression of the *Cd8a* gene in the colon. For both inoculums the HP diet increased the expression of the *Cd8a* gene in ileum. *Foxp3* expression (encoding FOXP3, the master regulator of Tregs) in colon was significantly higher in HM mice irrespective of diet. Together with the FACS results this may reflect the activation of immune dampening Tregs in response to the HM xenograft. This fact may be related to different LPS production from Gram-negative bacteria (Alexander and Rietschel, 2001) in the colon of HM mice. LPS is a microbial associated molecular pattern (MAMP), which through the stimulation of the Toll-like receptor 4 (TLR4) (Michie et al., 1988; Poltorak et al., 1998) stimulates the production of TNF-α, which is encoded by *Tnf*, which was higher expressed in colons of the HM mice. Hrncir et al. (2008) showed that LPS-rich diets drove expansion of Tregs (Hrncir et al., 2008), and we have previously shown that a cocktail of DSS, ampicillin and *E. coli* derived LPS given in early life induces the formation of Tregs (Bendtsen et al., 2017), which later in life reduces severity of oxazolone induced colitis in mice (Bendtsen et al., 2018). However, this increase in levels of FoxP3 + CD4+ positive cells was not accompanied by an increase in anti-inflammatory cytokines, such as IL-10 and TGFβ-1. In fact, *Tgf1b*, encoding TGFβ-1, which is crucial for induction, differentiation, and maintenance of peripheral Tregs (Fu et al., 2004; Tran, 2012), had lower expression in ileum from the HM mice, especially if fed the AS diet. In accordance with this, in HM mice *F. prausnitzii*, which is known to upregulate Tregs in both humans (Sedwick, 2014) and mouse models (Qiu et al., 2013), was not transferred successfully. However, HM mice had a high fraction of Tregs in the MLN, and compared to the other HM mice they had a lower abundance of *A. muciniphila*, which is another important inducer of Tregs (Derrien et al., 2017). Moreover, the HM mice in general, and especially those fed the AS diet, had significantly increased expression of *Il6*. IL6 is secreted by macrophages, when the TLR2-TLR6 complex is stimulated by MAMPs, such as peptidoglycan from Gram positive bacteria (Chen et al., 2006), which also may activate the signaling cascade pathway responsible for transcription of TNF-α (Takeuchi et al., 2002). However, IL-6 may also inhibit the LPS-induced TNF-α production (Inoue et al., 2007).

About DCs, the HM mice had higher fractions of CD103^–^ DCs in the MLN, while these were lower in their PP, where it was mostly due to a significantly higher fraction in MM mice on the AS diet. Accordingly, mice on the AS diet had significantly lower CD103^+^ tolerogenic DC’s in the PP. Interestingly, the expression of *Itgax*, i.e., the gene encoding the transmembrane protein CD11c on the DCs, was significantly higher in the ileum of MM mice, while it was significantly higher in the colon of HM mice. Thus, it could be different communication pathways dependent on tissue for members of the HM and MM, respectively.

Chung et al. (2012) found a very poor T cell response in HM colonized mice, which in their study were more comparable with GF mice. In our study, HM mice were only low on CD4^+^ T cells. Such differences between studies may be related to the human donor’s microbiota composition. For example, *Bacteroides*-derived LPS, which is the most common gut inhabitant in Finnish children, is a far weaker LPS inducer compared to the *E. coli-*derived LPS, which is the most common in Russian children, and may therefore explain differences between them in their Treg numbers and their risk of autoimmune disease (Vatanen et al., 2016). In a previous study of HM mouse transplantation, we used the same human donor material as in this study and observed major differences in gut immunological profile between HM and MM mice, but the MM originated from C57BL/6NTac mice at another Taconic barrier with segmented filamentous bacteria (SFB) (Lundberg et al., 2020). The MM of this study did not contain SFB’s, and although not alone driving immunological stimulation (Chung et al., 2012), SFB’s are very strong immunological modulators (Ivanov et al., 2009), and therefore the MM mice of this study may have been less immunologically mature compared to the MM mice in the previous study and hence less different from the HM mice. Not only ex-germ-free HM colonized mice, but also more ordinary SPF mice are often reported to be immunologically naïve and especially in lack of CD8^+^ T cells (Beura et al., 2016; Reese et al., 2016).

Although our study has some strengths due to the different dietary approaches, our study also has some limitations worth mentioning. The lack of reference values from gnotobiotic mice without inoculation triggers makes it impossible to associate changes in the gut immune response observed in the different groups fed with human diet to the changes in the gut microbiota. Moreover, differences in nutrient availability in each diet could also take part in the immune response itself. However, according to our results, chow diets should be reformulated for better colonization, at least in experiments where human microbiota is transplanted into mice. Thus, the current results will serve as a basis for future research.

## Conclusion

In conclusion, following the scientific literature, we confirmed that HM transplanting of GF mice leads to a much lower colonization efficiency and poorer generation of CD4^+^ Th cells. Colonization efficiency and immune stimulation in human microbiota transplanted mice could be moderately improved by feeding the mice a diet, in which the proportion of macronutrients are comparable to a human diet, although it will not raise the colonization efficiency and immune stimulation to a state comparable to a MM transplantation in mice.

## Data Availability Statement

Publicly available datasets were analyzed in this study. This data can be found here: NCBI SRA with the ID: PRJNA633958.

## Ethics Statement

The animal study was reviewed and approved by the Danish Animal Experimentation Inspectorate (Ministry of Environment and Food in Denmark) according to license no. 2012-15-2934-00256 and with the EU directive 2010/63/EU and the Danish Animal Experimentation Act (LBK 474 from 15/05/2014).

## Author Contributions

RL, AH, and IM-I conceived and designed the study. IM-I performed animal procedures, qPCR, DNA isolation, library building, FACS, and analyzed data. RL performed qPCR, helped on animal procedures and FACS, and analyzed data. CH guided and helped on FACS experiments and analyzes. LK guided DNA isolation and library building, and analyzed the 16S rRNA gene sequencing data. WK generated and pre-processed the sequencing data. SM guided the qPCR experiments and analyses. RL, IM-I, and AH drafted the manuscript. LK, CH, DS, AH, SM, WK, and DN critically revised and commented on the manuscript. All authors participated in data interpretation and approved the final version of the manuscript. IM-I had the final responsibility of the manuscript.

## Conflict of Interest

The authors declare that the research was conducted in the absence of any commercial or financial relationships that could be construed as a potential conflict of interest.

## Supplementary Material

FIGURE S1 Body weight, feed efficiency and long-term blood glucose of F1 mice with human or mouse microbiota. **(A)** Body weight from 3 week of age to 6 week of age. **(B)** Feed efficiency ratio. **(C)** Hba1c, or long-term blood glucose measured at 6 week of age. F1=offspring of originally colonized mice, i.e., born with the microbiota. HM, human microbiota; MM, mouse microbiota; AS, animal source diet; HP, human profile diet; C, control diet.

## Abbreviations

ADFC: Average daily food consumption
AS: animal source diet
C: control diet
DCs: dendritic cells
DSS: dextran sodium sulfate
F1: First generation
FOXP3: forkhead fox P3
HM: human microbiota
HP: human profile diet
LPS: lipopolysaccharide
MLN: mesenteric lymph nodes
MM: mouse microbiota
P: Parents’ generation
PP: Peyer’s patches
REGIII γ: regenerating islet-derived 3 gamma
SPF: specific pathogen free
TGF β -1: transforming growth factor beta-1
Th cells: T helper cells
TLR: toll like-receptors
Tregs: regulatory T cells.
