# Supplementary figures and images for: A Humanized Diet Profile May Facilitate Colonization and Immune Stimulation in Human Microbiota-Colonized Mice
Source: Front Microbiol. 2020 Jun 19;11:1336. doi: 10.3389/fmicb.2020.01336 (PMC7318556; doi:10.3389/fmicb.2020.01336)

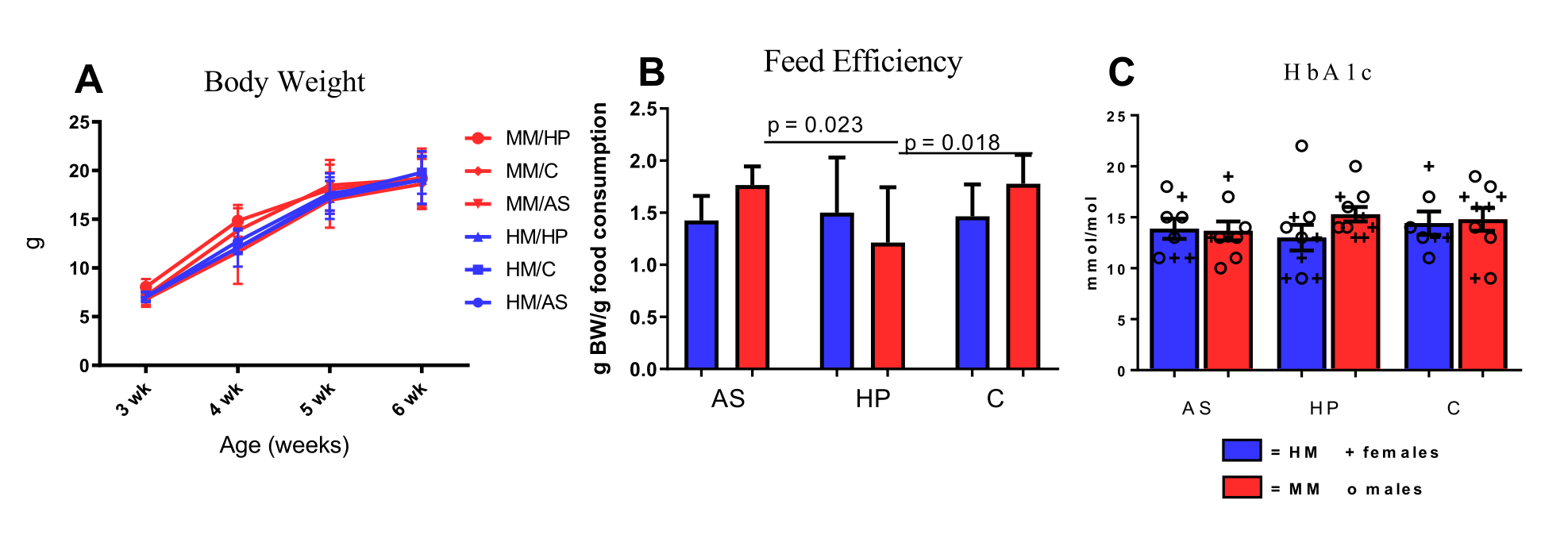

Supplement: FIGURE S1 — Body weight, feed efficiency and long-term blood glucose of F1 mice with human or mouse microbiota. (A) Body weight from 3 week of age to 6 week of age. (B) Feed efficiency ratio. (C) Hba1c, or long-term blood glucose measured at 6 week of age. F1=offspring of originally colonized mice, i.e., born with the microbiota. HM, human microbiota; MM, mouse microbiota; AS, animal source diet; HP, human profile diet; C, control diet. [file Image_1.TIFF]
