# Supplementary material for: A Humanized Diet Profile May Facilitate Colonization and Immune Stimulation in Human Microbiota-Colonized Mice
Source: Front Microbiol. 2020 Jun 19;11:1336. doi: 10.3389/fmicb.2020.01336 (PMC7318556; doi:10.3389/fmicb.2020.01336)
Supplement: Supplementary file 2 [file Table_1.DOCX]

| Table S1. **Composition of three diets used for the study of dietary impact on immune stimulation after inoculation with a human and a mouse microbiota in mice.** | | | | |
| --- | --- | --- | --- | --- |
|  | **Control (C)** | **Human Profile (HP)** | **Animal Source (AS)** | **Unit** |
| **Metabolic Energy** | 3200.50 | 3800.66 | 3200.50 | kcal/kg |
| **Crude nutrients** |  |  |  |  |
| Crude Protein | 192666.60 | 190800.02 | 192242.00 | mg/kg |
| Crude Fat | 43103.65 | 105284.25 | 43272.17 | mg/kg |
| Crude Fibre | 66145.04 | 31509.20 | 65756.40 | mg/kg |
| Crude Ash | 75836.35 | 45893.11 | 71691.35 | mg/kg |
| Moisture | 101540.39 | 96007.39 | 108611.39 | mg/kg |
| Disaccharide(s) | 25823.05 | 20699.05 | 25357.05 | mg/kg |
| Polysaccharide(s) | 372434.73 | 430708.43 | 370940.93 | mg/kg |
| **Amino acids** |  |  |  |  |
| Lysine | 4986.11 | 5480.71 | 11437.65 | mg/kg |
| Methionine | 3013.98 | 3055.08 | 5347.58 | mg/kg |
| Cystine | 3476.70 | 3520.00 | 3600.60 | mg/kg |
| Threonine | 6863.10 | 6784.50 | 6888.80 | mg/kg |
| Tryptophan | 2768.80 | 2657.50 | 2260.30 | mg/kg |
| Arginine | 11066.70 | 10990.50 | 9800.10 | mg/kg |
| Histidine | 4790.80 | 4800.00 | 4971.60 | mg/kg |
| Isoleucine | 8125.90 | 8314.50 | 7151.70 | mg/kg |
| Leucine | 12327.50 | 12664.50 | 14478.30 | mg/kg |
| Phenylalanine | 7427.90 | 7641.50 | 7658.30 | mg/kg |
| Valine | 6808.20 | 6559.50 | 5949.60 | mg/kg |
| Alanine | 9155.00 | 8990.00 | 5737.80 | mg/kg |
| Aspartic acid | 17142.60 | 17202.50 | 7480.50 | mg/kg |
| Glutamic acid | 43897.50 | 45980.00 | 32897.60 | mg/kg |
| Glycine | 8067.50 | 7593.00 | 5871.30 | mg/kg |
| Proline | 11703.30 | 12252.50 | 14069.70 | mg/kg |
| Serine | 9082.60 | 9199.00 | 6989.90 | mg/kg |
| Tyrosine | 6282.90 | 6421.00 | 7782.50 | mg/kg |
| **Vitamins** |  |  |  |  |
| Vitamin A | 15000.00 | 15000.00 | 15000.00 | IU/kg |
| Vitamin D3 | 600.00 | 600.00 | 600.00 | IU/kg |
| Vitamin E | 84.00 | 123.00 | 75.22 | mg/kg |
| Vitamin K3 | 3.00 | 3.00 | 3.00 | mg/kg |
| Vitamin B1 | 18.00 | 18.00 | 18.02 | mg/kg |
| Vitamin B2 | 12.00 | 12.00 | 12.18 | mg/kg |
| Vitamin B6 | 9.00 | 9.00 | 9.02 | mg/kg |
| Vitamin B12 | 0.02 | 0.02 | 0.03 | mg/kg |
| Nicotinic acid | 36.00 | 36.00 | 36.09 | mg/kg |
| Pantothenic acid | 21.00 | 21.00 | 21.06 | mg/kg |
| Folic acid | 3.24 | 3.25 | 3.20 | mg/kg |
| Biotin | 0.82 | 0.82 | 0.81 | mg/kg |
| Choline chloride | 1169.30 | 1078.50 | 1181.43 | mg/kg |
| Inositol | NA | NA | 6.05 | mg/kg |
| Vitamin C | 36.00 | 36.00 | 36.00 | mg/kg |
| **Minerals** |  |  |  |  |
| Calcium | 7351.88 | 7340.44 | 7285.43 | mg/kg |
| Available phosphorus | 5262.04 | 4702.11 | 5323.86 | mg/kg |
| Digestible phosphorus | 1505.00 | 2313.00 | 1698.50 | mg/kg |
| Magnesium | 1779.75 | 1142.75 | 1700.94 | mg/kg |
| Sodium | 3210.47 | 3238.51 | 2170.82 | mg/kg |
| Potassium | 4637.03 | 3437.41 | 4497.32 | mg/kg |
| Sulfur | 1865.50 | 1806.90 | 1966.30 | mg/kg |
| Chlorine | 3638.30 | 3524.55 | 3629.19 | mg/kg |
| Aluminium | 119.27 | 103.59 | 116.54 | mg/kg |
| **Trace elements** |  |  |  |  |
| Iron | 170.07 | 151.49 | 154.63 | mg/kg |
| Manganese | 67.35 | 50.73 | 65.88 | mg/kg |
| Zinc | 71.72 | 66.50 | 70.28 | mg/kg |
| Copper | 13.59 | 12.30 | 12.09 | mg/kg |
| Iodine | 1.38 | 1.38 | 1.41 | mg/kg |
| Molybdenum | 0.31 | 0.23 | 0.30 | mg/kg |
| Fluorine | 2.94 | 4.18 | 2.93 | mg/kg |
| Selenium | 0.24 | 0.21 | 0.28 | mg/kg |
| Cobalt | 0.65 | 0.74 | 0.66 | mg/kg |
| **Fatty acids** |  |  |  |  |
| Capric acid | NA | NA | 380.00 | mg/kg |
| Lauric acid | 15.00 | 80.00 | 460.00 | mg/kg |
| Myristic acid | 30.00 | 160.00 | 1720.00 | mg/kg |
| Palmitic acid | 2562.20 | 6392.00 | 2165.20 | mg/kg |
| Palmitoleic acid | 45.00 | 240.00 | 360.00 | mg/kg |
| Margaric acid | 30.00 | 160.00 | NA | mg/kg |
| Stearic acid | 962.60 | 2896.00 | 1749.60 | mg/kg |
| Oleic acid | 7256.20 | 25832.00 | 6485.20 | mg/kg |
| Linoleic acid | 16147.30 | 55628.00 | 7415.80 | mg/kg |
| Linolenic acid | 1090.50 | 1620.00 | 1133.00 | mg/kg |
| Arachidic acid | 15.00 | 80.00 | NA | mg/kg |
| Eicosenoic acid | 45.00 | 240.00 | NA | mg/kg |
| Behenic acid | 45.00 | 240.00 | NA | mg/kg |
| Butyric acid | NA | NA | 660.00 | mg/kg |
| Heptanoic (enanthic) acid | NA | NA | 580.00 | mg/kg |
| Octanoic (caprylic) acid | NA | NA | 200.00 | mg/kg |
| Erucic acid | 15.00 | 80.00 | 276.20 | mg/kg |
| Lignoceric acid | 75.00 | 400.00 | NA | mg/kg |
| **Total batch volume used for the study** | **10.00** | **10.00** | **10.00** | **kg** |
